# Slow freezing and 3D *in vitro* culture of prepubertal goat testes after short-term conservation

**DOI:** 10.1590/1984-3143-AR2025-0139

**Published:** 2026-07-20

**Authors:** Francisco Denilson Rodrigues Gomes, Lucy Vanessa Sulca Ñaupas, Gaby Judith Quispe Palomino, Rensson Homero Céliz Ygnácio, Naiza Arcângela Ribeiro de Sá, Anna Clara Accioly Ferreira, Marco Aurélio Schiavo Novaes, Gustavo Bezerra Nobre do Vale, Bruna Novaes Costa, Carolina Madeira Lucci, César Carneiro Linhares Fernandes, Davide Rondina, Alexandre Rodrigues Silva, José Ricardo de Figueiredo, Gildas Mbemya Tetaping, Ana Paula Ribeiro Rodrigues

**Affiliations:** 1 Laboratório de Manipulação de Oócitos e Folículos Ovarianos Pré-Antrais, Faculdade de Medicina Veterinária, Universidade Estadual do Ceará, Fortaleza, CE, Brasil; 2 Faculty of Medical Sciences, Private University of the East – UPE, Affiliate Ciudad del Este, Paraguay; 3 Laboratório de Reprodução Animal, Departamento de Ciências Fisiológicas, Instituto de Ciências Biológicas, Campus Universitário Darcy Ribeiro, Brasília, DF, Brasil; 4 Faculdade de Ciências da Saúde, Universidade de Fortaleza, Fortaleza, CE, Brasil; 5 Laboratório de Nutrição e Produção de Ruminantes, Universidade Estadual do Ceará, Fortaleza, CE, Brasil; 6 Laboratório de Conservação de Germoplasma Animal, Departamento de Ciência Animal, Universidade Federal Rural do Semi-Árido, Mossoró, RN, Brasil

**Keywords:** slow freezing, *in vitro* culture, testicles, male fertility, goat

## Abstract

This study This study aimed to evaluate two conservation times (2h vs 6h) at 4 ºC of prepubertal goat testicles for subsequent slow freezing and 3D in vitro culture. Testicular fragments from six animals were preserved at 4 ºC for 2 or 6 h. After 2 or 6 h of conservation, the fragments were randomly fixed, constituting the control (CTR2; CTR6), cultured in vitro (IVC2; IVC6), frozen (SFZ2; SFZ6), or frozen and cultured in vitro (SFZIVC2; SFZIVC6). All fragments were analyzed by classical histology (morphology), immunohistochemistry (PCNA), immunofluorescence (β-catenin), PCRq-RT (Oct4, C- kit, Bax, Bcl-2), transmission (TEM), and scanning (SEM) electron microscopy, respectively. The histomorphological data showed that regardless of the conservation time, whether followed by cryopreservation or not, epithelial (Ealt) and nuclear (Nalt) alterations increased after 48 h of IVC (p<0.05). Immunostaining for β-catenin was lower (p < 0.05) in SFZIVC2 and SFZIVC6 fragments compared with IVC2 and IVC6, respectively. Oct4 expression in SFZ6 was lower than that in CTR6 (p < 0.05). Conversely, C-kit expression was similar among CTR2, CTR6, SFZ2, and SFZ6 fragments. Bax and Bcl-2 expression were also similar among IVC2, IVC6, SFZIVC2, and SFZIVC6 fragments. The Bax/Bcl-2 ratio in IVC2 and SFZIVC6 was higher than that observed in CTR2 and SFZ6, respectively. In conclusion, our results demonstrate that controlling the duration of testicular storage at 4 °C before slow freezing and/or in vitro culture directly affects the viability and quality of testicular tissue, thereby influencing the success of reproductive function preservation.

## Introduction

Studies on human reproduction have shown that cryopreservation of immature testicular fragments is a promising and innovative biotechnology for preserving male fertility, especially in prepubertal individuals undergoing gonadotoxic treatments (Picton et al., 2015; Nmetou et al., 2019). Regarding animal reproduction, the expectations for this biotechnology have also been very positive. It aims to preserve genetic material, especially from endangered wildlife species (Silva et al., 2021), or even genetically and/or commercially valuable domestic species, such as small ruminants.

Previous studies have demonstrated the restoration of spermatogenesis in rabbits after fragments of testicles were frozen/thawed and grafted onto the tunica albuginea of mice. After two months of transplantation, mature spermatozoa were recovered from the fragments and used for *in vitro* fertilization of oocytes, resulting in the birth of live offspring (Shinohara et al., 2002). In pigs, frozen/thawed testicular fragments were xenotransplanted into the dorsal skin of mice for 11 months (Kaneko et al., 2013). In non-human primates, autologous transplantation was performed on the scrotal skin for 9 months (Fayomi et al., 2019). After recovery of the grafts, intracytoplasmic sperm injection (ICSI) was performed, resulting in the birth of offspring in pigs and non-human primates. Although these results are very promising, in production animals, transplantation is not always the best option, as maintaining a transplanted animal in the herd has a low cost-benefit ratio and requires specialized care. In this case, the *in vitro* culture of testicular tissue (IVC) after cryopreservation can be an excellent alternative and a trend in reproductive biotechnology to restore spermatogenesis and male reproductive function, even after the death of the individual (Portela et al., 2019).

Testicular fragments can be cultured *in vitro* in a two-dimensional (2D) or three-dimensional (3D) system. In the 2D system, the tissue is placed on an extracellular matrix (ECM) or directly in the well of a plate and completely covered with the culture medium. However, this system is suitable only for short-term *in vitro* culture (7 to 14 days on average), as it cannot mimic the natural environment to allow cell-cell and cell-matrix interactions similar to those that occur in vivo (Ghorbani et al., 2022). In the 3D system, the fragment is embedded in an ECM, which provides structural and biochemical support, allowing for better cell organization, maintenance of functionality, and interactions between cells and the environment, thus mimicking in vivo conditions (Alves-Lopes and Stukenborg, 2018). Recently, a study conducted by our team demonstrated that the *in vitro* culture of ovine testicular fragments embedded in an agarose gel matrix (3D system) resulted in less cellular loss and basal membrane rupture, suggesting tissue maintenance and protection (Celiz et al., 2023).

Depending on the environment the animal is in at the time the testicles are collected, the length of time they are stored during transportation is a factor that can negatively influence the ability of the cells and tissue to withstand the manipulations and procedures before cryopreservation and *in vitro* culture (Onofre et al., 2016). This period, when inadequate, directly affects the resumption of sperm cell metabolism after culture (Thuwanut et al., 2013), severely compromising the success of the technique. Since many animals live far from laboratories specialized in assisted reproduction techniques, defining the appropriate conservation period is crucial to ensure the success of cryopreservation and subsequent in vitro culture. Thus, the present study aimed to evaluate two conservation times (2 hours versus 6 hours) at 4 ºC for prepubescent goat testicles for subsequent slow freezing and 3D *in vitro* culture.

## Methods

### Ethical approval and chemicals used

This study was approved and performed under the guidelines of the Ethics Committee for Animal Use of the State University of Ceará (N° 08623404/2021).

Unless mentioned otherwise, all chemicals were purchased from Sigma Aldrich Co. (St. Louis, MO, USA), and the cryoprotective agents [ethylene glycol (EG) and dimethylsulfoxide (DMSO)] were obtained from Dinâmica (Diadema, SP, Brazil).

### Origin of animals and collection of testicles

Six pairs of testes from pre-pubertal mixed-breed goats (2 to 6 months old) were obtained through bilateral orchiectomy at the Dr. Esau Accioly Vasconcelos Agricultural Experimentation Farm, located in Guaiúba, CE. After collection, both pairs of testicles were washed once in 70% alcohol and twice in saline solution (0.9% NaCl) supplemented with antibiotics (penicillin 100 μg/mL and streptomycin 100 μg/mL), as described by Thuwanut et al. (2013). The samples were then transported to the laboratory at 4 °C for 2 hours, ensuring the immediate onset of cooling. In the laboratory, one testis was dissected, and the resulting fragments were allocated to the different experimental conditions after 2 hours of storage at 4 °C. The other testis was maintained at 4 °C for an additional 4 hours, then dissected, and its fragments were allocated to the experimental conditions after a total of 6 hours of storage.

### Experimental design

In the laboratory, testes previously preserved for 2 or 6 h were sectioned into blocks of approximately 1 cm, which were placed on a millimeter paper template to obtain standardized fragments of approximately 5 mm^3^. From each testis, 16 fragments were obtained and randomly allocated to the experimental groups. The fragments obtained from the testes of each animal preserved for 2 h were randomly distributed into the following groups: control 2 h (CTR2; n = 4), in vitro culture (IVC2; n = 4), slow freezing (SFZ2; n = 4), and freezing followed by in vitro culture (_SFZ_IVC2; n = 4). The procedure was repeated similarly for the fragments preserved for 6 h (CTR6; n = 4, IVC6; n = 4, SFZ6; n = 4, and _SFZ_IVC6; n = 4). The procedures were repeated six times using a total of 192 fragments, which were subjected to classical histology (hematoxylin and eosin staining; HE) (n = 48), immunofluorescence (β-catenin) and immunohistochemistry (PCNA) (n = 48), gene expression analyses (Oct4, C-kit, Bax, and Bcl-2) (n = 48), and transmission electron microscopy (TEM) and scanning electron microscopy (SEM) (n = 48).

### Slow freezing and thawing of testicular fragments

The Slow freezing was performed according to the protocol described by Pukazhenthi et al. (2015). Briefly, the testicular fragments were exposed to 1 mL of cryoprotectant solution (MEM-HEPES + 20% fetal bovine serum (FBS) and 20% dimethyl sulfoxide (DMSO) in 2.0 mL cryotubes at room temperature (RT: ~25 °C) for 10 minutes. Then, the cryotubes were transferred to Mr. Frosty (Thermo Fisher Scientific) containing isopropyl alcohol and maintained overnight in an ultra-freezer (-80°C). The following day, the cryotubes were removed and stored in liquid nitrogen (-196°C).

After 7 days, the cryotubes were thawed by exposure to room temperature (RT) for 1 min, followed by immersion in a water bath at 37 °C for 30 s. The testicular fragments were then washed twice in 0.5 mL of MEM-HEPES supplemented with 20% fetal bovine serum (FBS) for 4 min each.

### In vitro culture of testicular fragments

The fragments were cultured in vitro using a three-dimensional (3D) system, i.e. embedded in agarose gel, as previously described by Celiz et al. (2023). Briefly, agarose (1.5%, w/v; Sigma A6013-25G) was diluted in Milli-Q water and sterilized by autoclaving (2 atm, 121°C, 20 min). Subsequently, the agarose (1.5%) was diluted in a minimal essential medium (α-MEM; 8042) to a final concentration of 0.35%. Subsequently, all the fragments were inserted separately (equidistant by ~20 mm) into the agarose solution and kept at 4°C for 15 minutes to solidify. The agarose was cut into blocks (10 x 15 x 5 mm) containing a testicular fragment and cultured individually for 48 hours in 24-well plates containing 1 mL of α-MEM (Sigma-Aldrich, M8042) supplemented with ITS (10 µg/mL insulin, 5.5 µg/mL transferrin, 5 ng/mL selenium), glutamine (2 mM), hypoxanthine (2 mM), pyruvate (1 mM), rbFSH (10 ng/mL), LH (10 ng/mL), and 10% FBS at 34°C, 6% CO2 in the incubator (Singh et al., 2019).

### Histomorfological analysis

Fragments from all experimental conditions were fixed in Davidson's solution (22.2% formaldehyde, 33.4% ethanol, 11% glacial acetic acid, and 33.4% distilled water) at room temperature for 12 h. The samples were then dehydrated through a graded series of ethanol, cleared in xylene, embedded in paraffin, and sectioned at a thickness of 7 µm. They were stained with hematoxylin and eosin (H&E) for morphological evaluation and to assess the integrity of the seminiferous tubules (ST), as described in our previous study (Gomes et al., 2023). The STs were classified according to epithelial changes (Ealt: membrane retraction) and nuclear changes (Nalt: nuclear condensation) and assigned scores from 0 to 5, where 0: no changes; 1: <20%; 2: >20-40%; 3: >40-60%; 4: >60-80%; 5: >80%.

### Immunohistochemical analysis

For this analysis, fragments from all experimental conditions were fixed in 4% paraformaldehyde, dehydrated through a graded series of ethanol, cleared in xylene, embedded in paraffin, and sectioned at a thickness of 5 µm. Then, the sections were mounted on positively charged slides and processed for immunohistochemistry to detect the proliferating cell nuclear antigen (PCNA). For antigen retrieval, the slides were incubated in a specific pH protein retrieval buffer (Dako, Santa Clara, CA, USA) for 5 minutes at 80°C. Endogenous peroxidase activity was blocked using 10% hydrogen peroxide in methanol. The slides were incubated with primary antibody (1:600, anti-PCNA, Abcam Inc., Cambridge, MA, USA) for 30 minutes, followed by incubation with secondary antibody (goat anti-rabbit IgG) for the same period (1:200; Abcam Inc.). They were then incubated for 30 minutes with an avidin-biotin enzyme complex (ABC; Vector Laboratories, Burlingame, CA, USA) for reaction with 3,3'-diaminobenzidine chromogenic solution (DAB; Dako, Inc., USA) and counterstained with hematoxylin and 0.5% ammonia solution (Céliz et al., 2023). Mouse spleen fragments were used as positive controls, and sections processed without the primary antibody were considered negative controls. For the quantification of PCNA‑positive immunolabeled cells, a total of 30 seminiferous tubules per experimental group were randomly selected across all fragments within that group.

### Immnunofluorescence analysis

The testicular fragments were processed as described for immunohistochemistry. However, antigen retrieval was performed in 0.01 M sodium citrate buffer (pH = 6.0) at 95-100°C for 5 min. Non-specific blocking was performed by incubating the slides in PBS containing 1% BSA for 1 h at room temperature. The slides were then incubated overnight at 4°C with rabbit anti-β-catenin antibody (1:400 - ab16051; Abcam Inc.). After this step, the samples were washed twice in PBS and incubated with Alexa Fluor® 488-conjugated secondary antibody (1:200) for 60 min at room temperature. The slides were then mounted with 4',6-diamidino-2-phenylindole (DAPI - ab104139, Abcam Inc.) and the sections were evaluated using confocal microscopy (Zeiss LSM 700META, Weimar, Germany). The negative control was performed in the absence of the primary antibody. β‑catenin immunostaining was evaluated in a total of 30 seminiferous tubules per experimental group, randomly selected across all tissue fragments belonging to each group, and image analysis was performed using ImageJ software (version 1.54a; NIH, Bethesda, MD, USA) through pixel-based analysis of the regions of interest, with the mean intensity values obtained being directly used for statistical analysis between the experimental groups.

### RNA extraction and real-time PCR (qPCR)

To evaluate the levels of gene expression for Oct4, c-Kit, Bax, and Bcl-2, the testicular fragments were subjected to total RNA extraction using the Trizol reagent (Invitrogen, Carlsbad, CA, USA), following the manufacturer's recommendations. After extraction, the RNA concentration was determined using the NanoDrop system with 2 μL of the material. Total RNA purification was carried out with the PureLink™ RNA Mini Kit (Ambion®, Carlsbad, CA, USA), according to the manufacturer's recommendations. Before complementary DNA (cDNA) synthesis, all samples were standardized with the same amount of RNA to minimize variability during qPCR. The cDNA was synthesized from the isolated RNA using Superscript III RT-PCR (Invitrogen, Carlsbad, CA, USA).

The primers were designed to amplify the mRNA levels for Oct4, C-Kit, Bax, and Bcl-2 (Table 1). As an endogenous control, the gene glyceraldehyde-3-phosphate dehydrogenase (GAPDH) was used for normalization, gene expression assessment, and evaluation of gene expression stability in all samples. The thermocycling profile for the first qRT-PCR cycle was as follows: initial denaturation and polymerase activation for 15 min at 94°C, followed by 40 cycles of 15s at 94°C, 30s at 60°C, and 45s at 72°C. The final extension was 10 minutes at 72°C. The reactions were performed using an IQ5 real-time PCR detection system (Eppendorf, Hamburg, Germany, USA). The transformation of threshold cycle (Ct) values into normalized relative mRNA expression levels was carried out using the delta-delta-Ct method.

**Table 1:** Oligonucleotide primers used for PCR analysis of goat testicular tissues.

Target Gene	Primer sequence	Bp	Number of access	Reference
*C-kit*	F: GAATAGCTGGCATCAGGGTG	20	AF263827.1	Binsila et al. (2018)
	R: CCAGATCCACATTCTCTCCATC	22		
*Oct4*	F: GAGGAGTCCCAGGACATCAA	20	XM012101009	Bebbere et al. (2019)
	R: CCGCAGCTTACACATGTTCT	20		
*Bax*	F:TTTTGCTTCAGGGTTTCATCCAGGA	25	NM173894	Gomes et al. (2023)
	R: CAGCTGCGATCATCCTCTGCAG	22		
*Bcl2*	F: GTTTTCCGACGGCAACTTC	19	JN036558.1	Gomes et al. (2023)
	R: GGATGGTCCTGATCAACTCG	20		
*GAPDH*	F: ATGCCTCCTGCACCACCA	18	298676424	Ferreira et al. (2018)
	R: AGTCCCTCCACGATGCCAA	19		

F: Forward primer; R: Reverse primer.

### Scanning and transmission electron microscopy analysis

Testicular fragments were fixed with Karnovsky's fixative overnight at 4°C and subsequently stored in 0.1 M sodium cacodylate buffer (pH 7.2) until processing.

For scanning electron microscopy, the samples were subsequently post-fixed in 2% osmium tetroxide for 1 h and then washed in distilled water (twice). The samples were then dehydrated in increasing acetone series (30%, 50%, 70%, 90%, 100%, 100%), critical-point dried with CO2, and mounted on stubs using carbon tape. The mounted samples were gold-coated and observed under a scanning electron microscope (Jeol, Japan) at 5.0 kV.

For transmission electron microscopy analysis, tissue fragments (1 mm^3^), a size considered ideal for adequate fixation, reagent penetration, and preservation of tissue ultrastructure, were used. The samples were post-fixed with 1% osmium tetroxide and 1.6% potassium ferrocyanide for 1 hour and then washed three times in distilled water. Subsequently, block staining was performed with 0.5% uranyl acetate overnight at 4°C. The following day, the samples were washed again in distilled water and dehydrated in increasing acetone series (30%, 50%, 70%, 90%, 100%, 100%) at 20-minute intervals for each. After infiltrating the samples in spur resin, they were embedded in pure resin, mounted in silicone molds, and placed in an incubator (60 - 65ºC) for 3 days to allow the resin to polymerize. The polymerized resin blocks were sectioned using an ultramicrotome (Leica EM UC7, Leica Microsystems, Wetzlar, Germany) to obtain semi-thin sections (3 µm) for identifying the area of analysis, and subsequently to obtain ultra-thin sections (60 nm) for final sample examination. The ultra-thin sections were collected on copper grids and examined under a transmission electron microscope (Jeol JEM 1011, Japan) at 80 kV for analysis of germline cells, Sertoli cells, Leydig cells, and cellular organelles.

### Statistical analysis

The analyses were conducted using the Statistical Package for the Social Sciences (SPSS version 27.0). Initially, the data were analyzed using the Shapiro-Wilk normality test, which indicated a predominantly non-normal distribution for the various variables under study. Therefore, comparisons were made using a nested design with the Mann-Whitney test, and results were considered significant when p<0.05.

## Results

### Histomorphological analysis

Table 2 shows that epithelial changes (Ealt) increased significantly in the SFZ2, SFZ6, and SFZIVC2 treatments compared to the CTR2, CTR6, and IVC2. The nuclear changes (Nalt) in the SFZ2 and SFZIVC2 treatments also increased significantly compared to the CTR2 and IVC2, respectively.

**Table 2:** Histomorphological evaluation of goat testicular tissue preserved for 2h (CTR2 – fresh; IVC2 – fresh cultured; SFZ2 – slow freezing; _SFZ_IVC2 – frozen cultured) or 6h (CTR6, IVC6, SFZ6, _SFZ_IVC6), frozen, followed or non-cultures by in vitro culture.

**Alterations**	**Conservation time**	**Non-cultured Testis**		**Cultured testis**	
		**CTR**	**SFZ**	**IVC**	***_SFZ_*IVC**
**Epithelial alterations (Ealt)**	2 h	0.0 ± 0.0 ^Aa^	0.15 ± 0.05 ^Ba^	1.12 ± 0.10 ^Aa*^	2.55 ± 0.07 ^Ba^*
	6 h	0.33 ± 0.08 ^Aa^	0.50 ± 0.09 ^Bb^	2.82 ± 0.08 ^Ab*^	2.70 ± 0.09 ^Aa*^
**Nuclear alterations (Nalt)**	2 h	0.08 ± 0.04 ^Aa^	0.30 ± 0.08 ^Ba^	2.56 ± 0.18 ^Aa*^	3.28 ± 0.13 ^Ba*^
	6 h	0.17 ± 0.05 ^Ab^	0.20 ± 0.05 ^Aa^	2.92 ± 0.07 ^Ab*^	2.98 ± 0.08 ^Ab*^

^A-B^Indicates differences between control and slow freezing within the non-cultures or cultured group; ^a-b^Indicates differences between conservation times in the control or slow freezing treatment. ^*^Indicates differences between the non-cultured and cultured groups subjected to the same treatment (control or slow freezing).

The comparison between the conservation times (2h vs. 6h) revealed that the treatments after 6 h (SFZ6 and IVC6) had more Ealt than those after 2 h (SFZ2 and IVC2). However, no significant differences were observed between _SFZ_IVC2 and _SFZ_IVC6. In the control and after *in vitro* culture, the Nalt were more marked (p < 0.05) after 6 h (CTR6 and IVC6) than after 2 h (CTR2 and IVC2) of conservation. In contrast to what was found for the Ealt, surprisingly, the Nalt were more pronounced (p < 0.05) in _SFZ_IVC2 than in _SFZ_IVC6.

**Figure 1:**
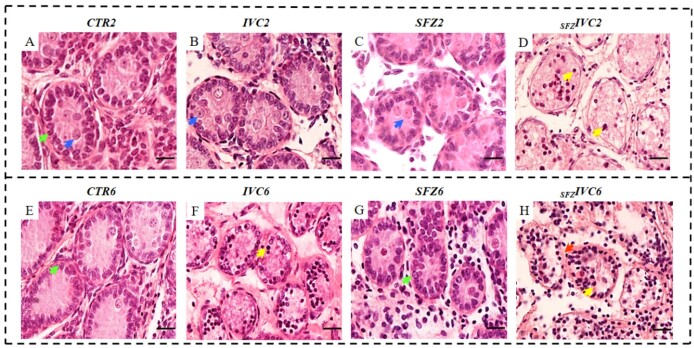
Sections of goat testicular tissue stained with Hematoxylin - Eosin (HE). (A, C, E, and G) Morphology of seminiferous tubules observed in the treatments (CTR2, CTR6, SFZ2, and SFZ6) with well-preserved testicular parenchyma and nuclear structure, allowing a clear distinction between spermatogonia (green arrows) and Sertoli cells (blue arrow), as well as the organization of the cellular niche. (B, D, F, and H) Morphology of seminiferous tubules observed in the treatments (IVC2, IVC6, _SFZ_IVC2, and _SFZ_IVC6) showing pyknotic nuclei (yellow arrow) and nuclear shrinkage (red arrow). Scale bar 50 μm.

Finally, regardless of the time of conservation or freezing, epithelial and nuclear changes increased significantly after in vitro culture. Figures 1A-1H show histomorphological microphotographs of the different treatments.

### Cell proliferation evaluated by immunostaining for PCNA

**Figure 2:**
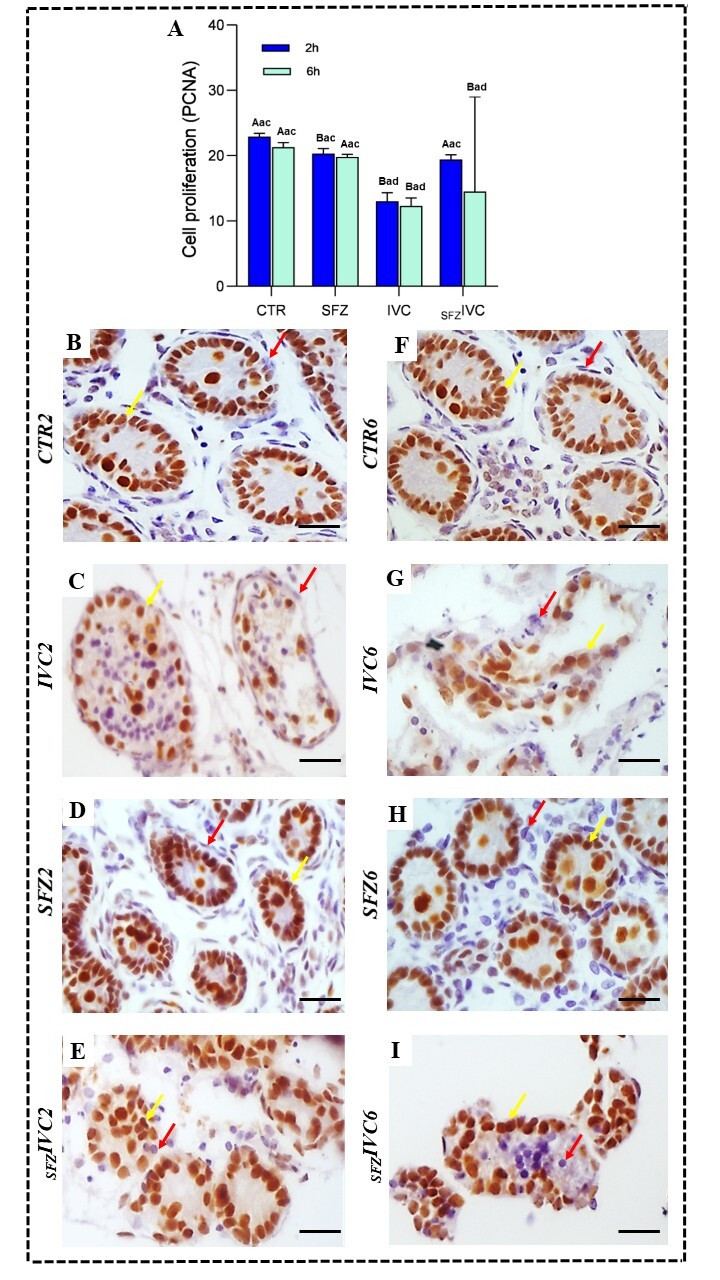
Immunostaining for PCNA protein in seminiferous tubules of prepubertal goat testes (A). Images of testicular fragments preserved for 2h (CTR2 – fresh; IVC2 – fresh cultured; SFZ2 – slow freezing; _SFZ_IVC2 – frozen cultured) (B-E). Testis preserved for 6h (CTR6 – fresh, IVC6 fresh cultured, SFZ6 – slow freezing, _SFZ_IVC6 – frozen cultured) (F-I). Yellow arrows indicate cells labeled for PCNA protein, while red arrows indicate unlabeled cells. Scale bar 50 μm. Different letters indicate statistically significant differences (p < 0.05). ^A,B^compare the control and slow-freezing treatments within the non‑cultured or cultured group. ^a, b^ compare the conservation times in the control or slow freezing treatment. ^c,d^ indicate differences between non‑cultured and cultured groups subjected to the same treatment (control or slow freezing).

In Figure 2A, the data show that after freezing, the cell proliferation in the testicular fragments preserved for 2 h (SFZ2) was lower (p < 0.05) than the control (CTR2); however, after 6 h of conservation (SFZ6), it was similar (p > 0.05) to the control (CTR6). On the other hand, after in vitro culture, cell proliferation in fresh (non-frozen) testicular fragments was lower than that in the control group after 2 and 6 h of preservation, respectively.

The comparison between the conservation times under different experimental conditions did not show significant differences in all treatments. After *in vitro* culture, cell proliferation in the fresh or non-frozen testicular fragments (IVC2, IVC6) was lower (p < 0.05) than the control at 2 (CTR2) and 6 (CTR6) h of conservation, respectively. On the other hand, after *in vitro* culture, although there was no significant difference after 2 h of conservation, in the samples preserved for 6 h and then frozen (_SFZ_IVC6), cell proliferation was lower (p < 0.05) than in the samples that were only frozen (SFZ6). Representative microphotographs of PCNA immunolabeling in goat testes are shown in Figure 2B-2I.

### Immunofluorescence for the β-catenin protein

**Figure 3:**
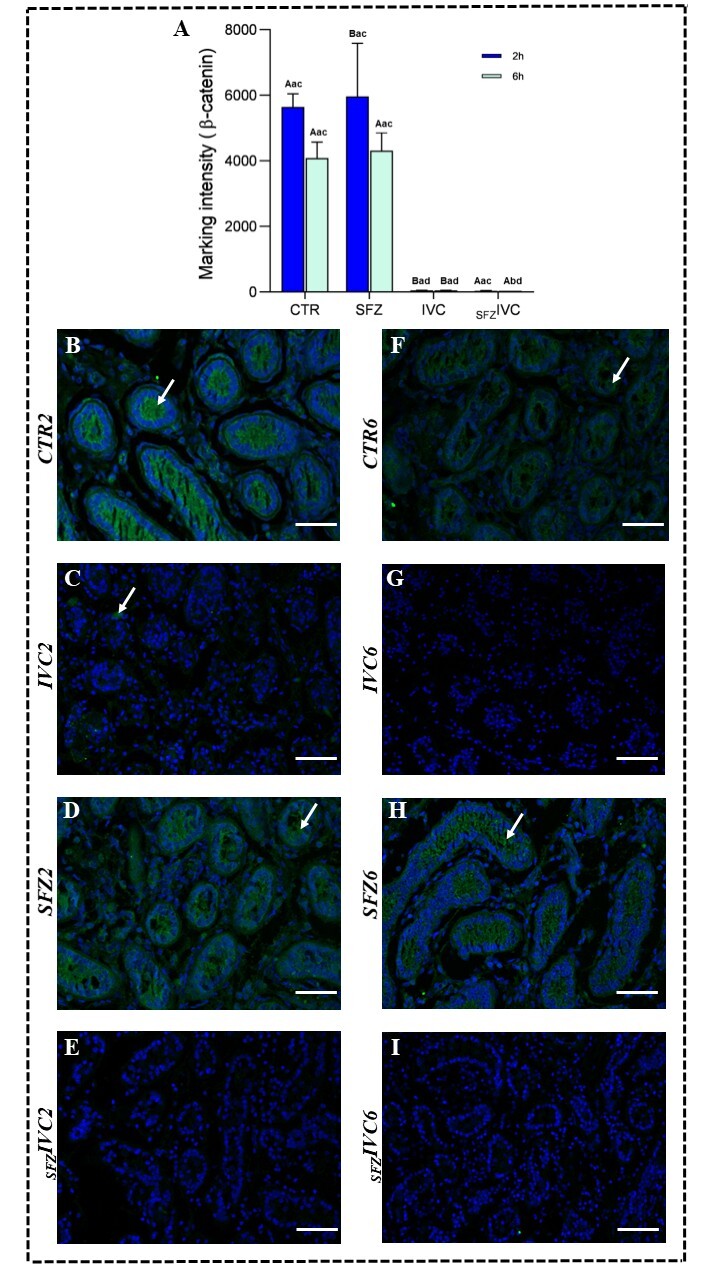
Immunofluorescence for β-catenin protein in seminiferous tubules of prepubertal goat testes (white arrow) (A). Images of testicular fragments preserved for 2h (CTR2 – fresh; IVC2 – fresh cultured; SFZ2 – slow freezing; _SFZ_IVC2 – frozen cultured) (B-E). Testicles preserved for 6h (CTR6 - fresh, IVC6 – fresh cultured, SFZ6 – slow freezing, _SFZ_IVC6 – frozen cultured) (F-I). Scale bar 50 μm. Different letters indicate statistically significant differences (p < 0.05). ^A, B^ compare the control and slow-freezing treatments within the non‑cultured or cultured group. ^a,b^compare the conservation times in the control or slow freezing treatment. ^c,d^indicate differences between non‑cultured and cultured groups subjected to the same treatment (control or slow freezing).

In Figure 3A, we can observe that the testicular fragments from the SFZ2 treatment showed higher fluorescence intensity (p < 0.05) for β-catenin than the CTR2, with no difference (p > 0.05) for these same treatments after 6 hours of conservation. On the other hand, after *in vitro* culture, the fluorescence intensity for this protein in the frozen fragments (_SFZ_IVC2, _SFZ_IVC6) was lower (p < 0.05) than in the fresh or non-frozen fragments (IVC2, IVC6). The comparison between conservation times (2h vs. 6h) under different conditions showed that the immunolabeling for β-catenin in the _SFZ_IVC6 treatment was higher (p > 0.05) than that observed for _SFZ_IVC2. After *in vitro* culture, the fluorescence intensity in the fresh treatments (IVC2, IVC6) or frozen treatment (_SFZ_IVC6) was lower than that in the control fragments (CTR2, CTR6) or those that were only frozen (SFZ6). Representative microphotographs of immunostaining for β-catenin in goat testes are shown in Figure 3B-3I.

### Gene expression pattern in caprine testis

**Figure 4:**
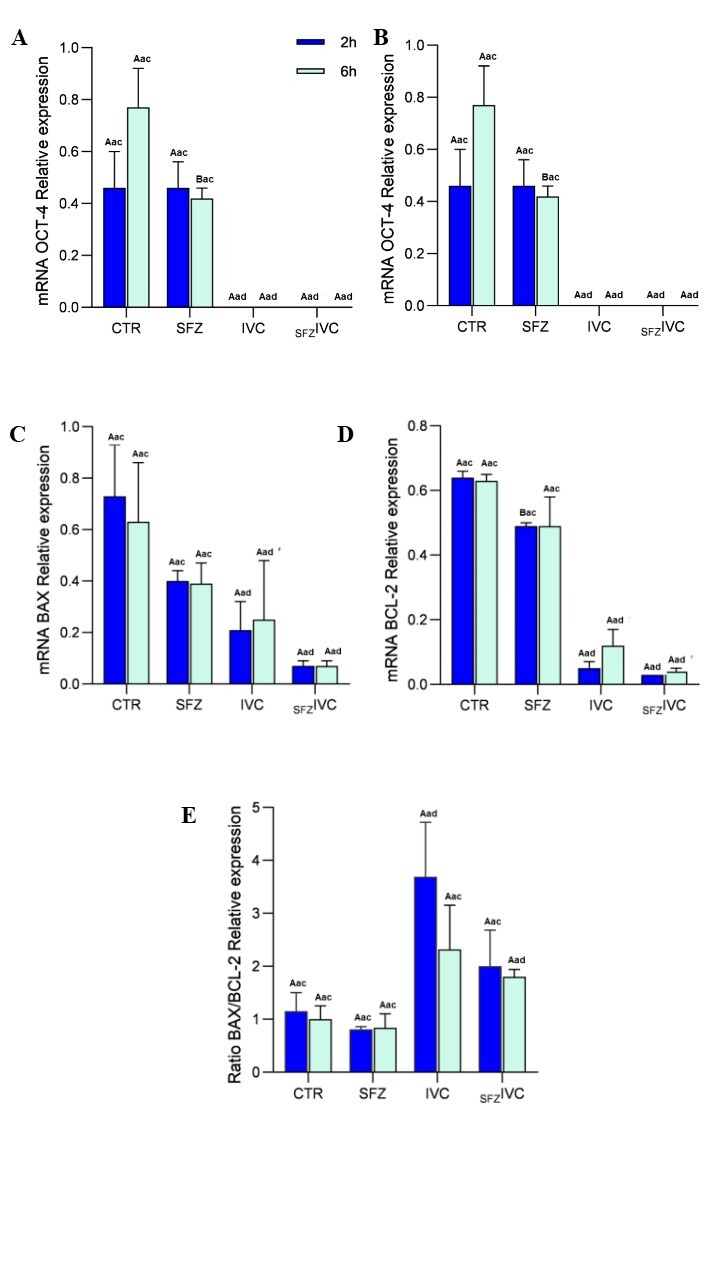
Relative expression of Oct4 (A), C-kit (B), Bax (C), Bcl-2 (D), and Bax/Bcl-2 ratio (E) in prepubertal goat testicles preserved for 2 h (CTR2 – fresh; IVC2 – fresh cultured; SFZ2 – slow freezing; _SFZ_IVC2 – frozen cultured) and testicles preserved 6 h (CTR6 - fresh, IVC6 – fresh cultured, SFZ6 – slow freezing, _SFZ_IVC6 – frozen cultured). Different letters indicate statistically significant differences (p < 0.05). ^A,B^compare the control and slow-freezing treatments within the non‑cultured or cultured group. ^a,b^compare the conservation times in the control or slow freezing treatment. ^c,d^indicate differences between non‑cultured and cultured groups subjected to the same treatment (control or slow freezing).

The data presented in Figure 4 revealed that the gene expression of Oct4 in SFZ6 was lower (p < 0.05) than that of the control (CTR6). After *in vitro* culture, the gene expression for Oct4 and C-kit in the treatments IVC2, IVC6, _SFZ_IVC2, and _SFZ_IVC6 was also lower (p < 0.05) than that found for the control (CTR2, CTR6), as well as for the fragments that were only frozen (SFZ2 and SFZ6).

The expression of the Bcl2 gene in the SFZ2 treatment was lower (p < 0.05) than in the respective control (CTR2). After *in vitro* culture, the expression of the Bax and Bcl2 genes in the treatments IVC2, IVC6, _SFZ_IVC2, and _SFZ_IVC6 was also lower (p < 0.05) than that observed in the non-cultured fragments (CTR2, CTR6, SFZ2, and SFZ6). Furthermore, the Bax/Bcl2 ratio in CTR2 and SFZ6 was lower than that observed for the treatments IVC2 and _SFZ_IVC6, respectively.

### Architecture of testicular tissue after freezing and/or in vitro culture

Scanning electron microscopy (SEM) showed that the architecture of the testicular parenchyma, as well as the cellular niche (Leydig cells, Sertoli cells, and spermatogonia), remained preserved in the testicular tissue that was only frozen (SFZ2 and SFZ6), similarly to the controls preserved for 2 or 6 hours (CTR2 and CTR6). Similarly, the analysis of fresh (non-frozen) fragments and fragments subjected only to freezing revealed no morphological alterations, with preservation of seminiferous tubule integrity and maintenance of the testicular parenchyma.

**Figure 5:**
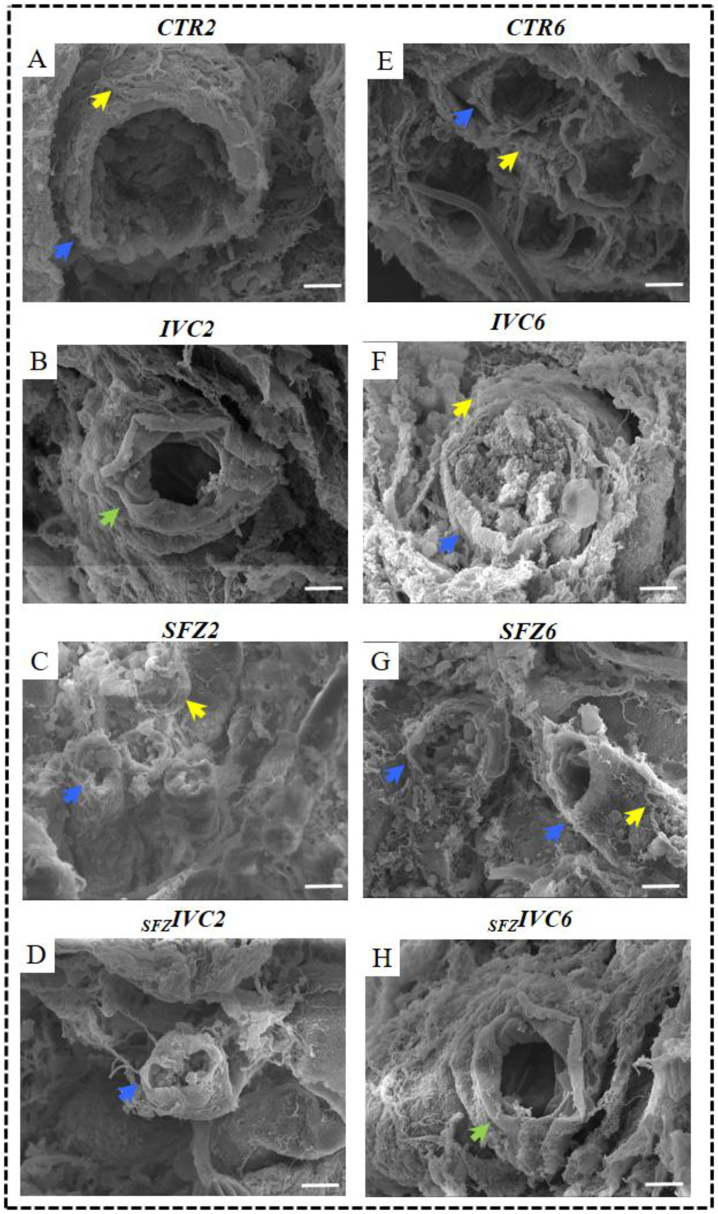
Scanning electron microscopy images of seminiferous tubules in prepubertal goat testicles. (A-D) Images of testicular fragments preserved for 2 hours (CTR2 – fresh; IVC2 – fresh cultured; SFZ2 – slow freezing; _SFZ_IVC2 – frozen cultured). (E-H) Testicles preserved for 6 hours (CTR6 - fresh, IVC6 – fresh cultured, SFZ6 – slow freezing, _SFZ_IVC6 – frozen cultured). The images show intact seminiferous tubules (blue arrow), Leydig cells (yellow arrow), and seminiferous tubules exhibiting cell loss (green arrow). Scale bar: 50 μm.

The testicles cultured *in vitro* before (IVC2, IVC6) or after slow freezing (_SFZ_IVC2 and _SFZ_IVC6) showed a reduction in diameter, disorganization, and a decrease in seminiferous tubules, as well as membrane shrinkage and loss of the extracellular matrix. Representative scanning electron microscopy images of goat testicles are shown in Figure 5.

**Figure 6:**
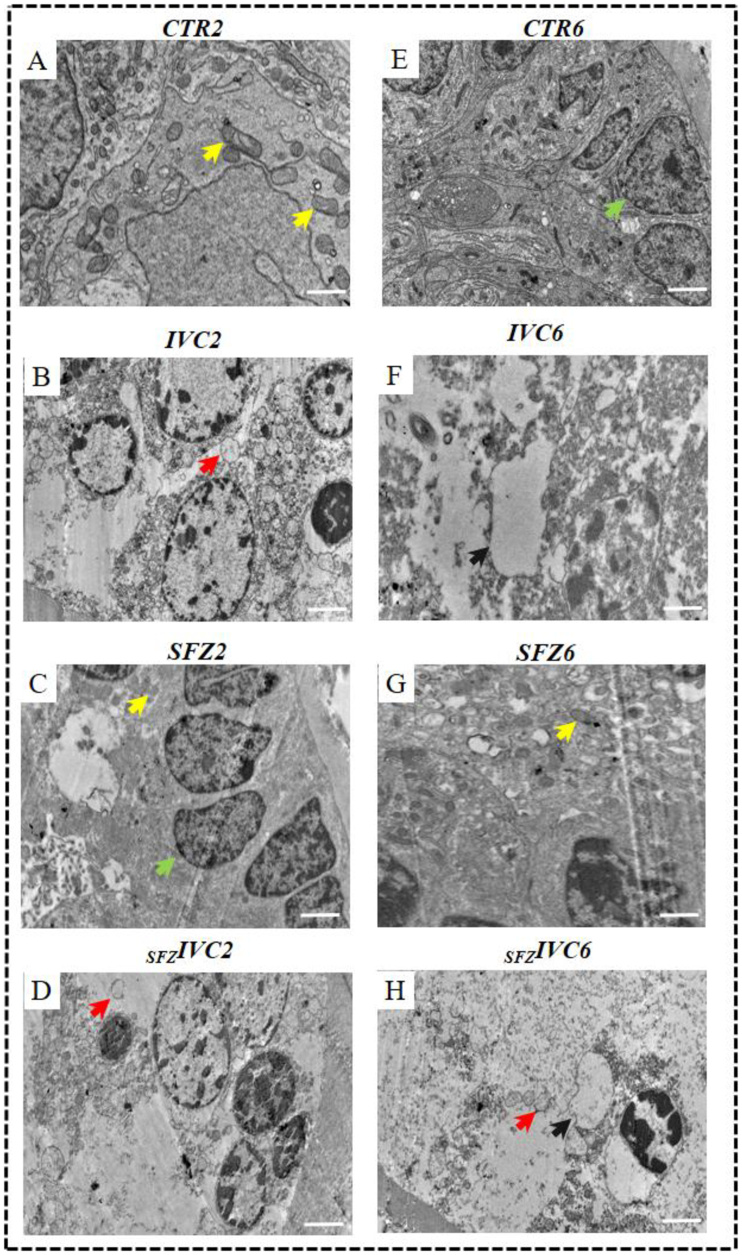
Transmission electron microscopy images of testicular tissue from prepubertal goats. (A-D) Images of testicular fragments preserved for 2 h (CTR2 – fresh; IVC2 – fresh cultured; SFZ2 – slow freezing; _SFZ_IVC2 – frozen cultured). (E-H) Testicles preserved for 6 hours (CTR6 - fresh, IVC6 – fresh cultured, SFZ6 – slow freezing, _SFZ_IVC6 – frozen cultured). The images show intact mitochondria (yellow arrow), preserved Sertoli cells (green arrows), swollen mitochondria (red arrow), and vacuoles (black arrow). Scale bar: 50 μm.

Additionally, transmission electron microscopy (TEM) allowed for the observation of nuclear membrane integrity, nuclear chromatin, organelles, and cytoplasmic vacuolization in the testicular fragments. The TEM revealed that after slow freezing (SFZ2 and SFZ6), cell conservation in the seminiferous tubules was similar to that observed in fresh testicles (CTR2 and CTR6), with the presence of Sertoli cells, spermatogonia, and normal mitochondria. Additionally, analysis of different preservation times within the same experimental condition did not reveal ultrastructural alterations in the evaluated cells. On the other hand, under experimental conditions after *in vitro* culture (IVC2, IVC6, _SFZ_IVC2, and _SFZ_IVC6), morphological alterations were observed, such as the presence of vacuolization, mitochondria with loss of ridges, and absence of cell delimitation. Representative transmission electron microscopy images of goat testicles are shown in Figure 6.

## Discussion

The success of cryopreservation and in vitro protocols for testicular fragments from sexually immature animals, among other factors, depends on the conservation time immediately after obtaining the testicles and their manipulation for cryopreservation itself, without compromising cellular and tissue integrity. Therefore, in this study, we investigated for the first time the effect of two conservation times of prepubertal goat testicles for 2 or 6 h at 4 ºC, with a view to slow freezing and in vitro culture in a 3D system.

The morphological data showed that after slow freezing (SFZ6) or *in vitro* culture (IVC6), the fragments preserved at 4 ºC for 6 h showed more epithelial changes than those maintained for 2 h (SFZ2 and IVC2). Previous studies have also reported that longer conservation periods at 4 ºC were detrimental to fragments of porcine (6 days) (Yang et al., 2010), feline (5 days) (Mota et al., 2012), and bovine (2 days) (Tang et al., 2024) testicles, while conservation for approximately 2 h was able to preserve the testicular parenchyma of goats (Patra et al., 2021) and sheep (Singh et al., 2019; Celiz et al., 2023). Prolonged conservation periods can cause hypoxia and compromise energy production (ATP) in cells due to mitochondrial dysfunction (Flood et al., 2023). This directly affects energy metabolism, limiting the cell´s ability to carry out vital processes. In the context of prepubertal goat testes, extending cold ischemia from 2 h to 6 h intensifies energy debt, acidosis, and disruption of epithelial junctions, thereby exacerbating oxidative stress upon reoxygenation and weakening the blood–testis barrier. This mechanism may account for the greater structural susceptibility observed at 6 h and supports the preference for a 2‑h preservation period to maintain the integrity of the seminiferous niche (Dutta et al., 2021). Another factor to be considered is the freezing method adopted in this study, which consisted of an uncontrolled slow-freezing approach (*Mr. Frosty*). The absence of a programmable system to regulate the cooling rate may have increased intracellular ice crystal formation and promoted osmotic damage, resulting in greater structural disorganization of the tissue after freezing and, notably, after in vitro culture, when compared with controlled protocols that provide greater precision and enhanced cellular protection (Meneghel et al., 2020).

The morphological analysis also showed that, regardless of the conservation time, whether followed by cryopreservation or not, the Ealt and Nalt increased significantly after 48 hours of IVC. Previous studies have also reported an increase in morphological alterations in cryopreserved testicular fragments from cats (Macente et al., 2022) and black-footed ferrets (Lima et al., 2020) following 24 h of in vitro culture in an agarose matrix. This effect may be due to the reduction of oxygen, changes in osmolarity, and pH that occur during *in vitro* culture, affecting the compartments of the seminiferous tubule epithelium and consequently cell viability (Ogawa et al., 2024). Considering these changes that occur naturally during *in vitro* culture, it is believed that the 3D IVC system is promising for restoring the function of testicular cells, especially when combined with the addition of supplements such as Knockout Serum Replacement (KSR), hormones, growth factors, and antioxidants (Ibtisham et al., 2023). In addition, it is possible that the agarose matrix used in this study contributed to the increase in observed alterations, as its physical properties, although widely employed in three-dimensional culture systems, may affect the diffusion of nutrients and essential factors depending on the experimental conditions. According to Yarnpakdee et al. (2022), the hydrogen bonds present in the agarose matrix hinder the perfusion of the medium, minimizing nutrient absorption and negatively affecting tissue architecture and function. Previous studies have shown that matrix sources such as collagen (Lee et al., 2006; Lee et al., 2007), Matrigel (Oliver and Stukenborg., 2020), dextran (Tan et al., 2024), and alginate (Tan et al., 2024) preserved the integrity of the seminiferous tubule and the presence of a high density of spermatocytes in murine testicular fragments cultured *in vitro*.

In our study, we realized immunolocalization for PCNA (Proliferating Cell Nuclear Antigen) to quantify spermatogonia and primary spermatocytes in proliferation. The results showed no alterations in any of the treatments between conservation times. Recently, a study carried out by our team on adult sheep testicles also showed that PCNA labeling was similar between non-cultured and *in vitro* cultured fragments (Celiz et al., 2023). Similar results have been reported in goat testicles after cryopreservation and *in vitro* culture for 14 days (Patra et al., 2021). Due to their germinative nature and biological and physiological characteristics, spermatogonia and spermatocytes can resist osmotic and thermal stress and have a high capacity for cellular repair (Celino et al., 2011; Ibtisham et al., 2017). This may explain the results obtained in our study. In addition, previous studies in rats have shown that variations in testicular preservation time, within controlled intervals, do not alter the quantification of cell proliferation (Bryant and Boekelheide, 2007; Elfageih et al., 2023).

We also investigated the protein β-catenin, which is involved in Wnt/β-catenin signaling and is crucial for the development of germ cells (spermatogonia, spermatocytes, and spermatids) and somatic cells (Sertoli and Leydig cells), as reported by several authors (Tanwar et al., 2010; Dong et al., 2015; Takase and Nusse., 2016). The results showed that regardless of the conservation time (2h vs 6h), after IVC, the immunolabeling for β-catenin in the previously frozen fragments was significantly lower than that observed for fresh fragments. It appears that cryopreservation may be related to the decrease in the expression of this protein, which could compromise spermatogenesis, disorganize the seminiferous epithelium, and affect cellular signaling, crucial for testicular development and function (Chang et al., 2011). Cryopreservation is known to cause endoplasmic reticulum stress, leading to reduced stability of β-catenin (Xia et al., 2019). Additionally, the freezing/thawing process can impair pathways such as Wnt/catenin, negatively affecting the survivability of germ cells (Yao et al., 2020). Furthermore, changes in β-catenin levels can lead to decreased cell proliferation and increased oxidative stress (Catalano et al., 2021).

Gene expression for Oct4 and C-kit was similar between fresh and frozen fragments after 2 h or 6 h of conservation. Recently, we obtained similar results in testicular fragments from prepubertal goats, previously frozen (Gomes et al., 2023). Oct4 is essential for maintaining the pluripotency and self-renewal of germ cells, as well as regulating the expression of critical genes for the development and differentiation of germ cells (Shi and Jin., 2010). C-kit, on the other hand, is also expressed early during spermatogenesis and is important for the survival, proliferation, and differentiation of germ cells, as well as for the cellular signaling that promotes spermatogenesis (Zhang et al., 2011). On the other hand, OCT4 expression in the SFZ6 treatment was lower than that observed in CTR6. The decrease in the expression of these genes can be explained by the stress accumulated during the prolonged conservation time and the subsequent cryopreservation process. The decrease in Oct-4 may indicate that the germ cells have lost their pluripotent state and have initiated differentiation (Sajini et al., 2012).

Except for Bcl-2 expression in the SFZ2 treatment, which was lower than that observed in the corresponding control (CTR2), the expression of the pro-apoptotic gene Bax and the anti-apoptotic gene Bcl-2 was comparable between fresh and cryopreserved fragments, as well as after in vitro culture, regardless of the conservation time. Bax and Bcl-2 are important proteins involved in the regulation of apoptosis, or programmed cell death, which is crucial for maintaining cellular homeostasis in various tissues, including the testes (Qian et al., 2022). The Bax/Bcl-2 ratio after IVC2 and _SFZ_IVC6 was higher than in CTR2 and SFZ6, respectively. Hajiaghalou et al. (2016) reported that pre-pubertal mouse testicles previously cryopreserved and cultured for a short period (20 h) also showed a higher Bax/Bcl-2 ratio than fresh testes. The balanced regulation between pro-apoptotic and anti-apoptotic proteins ensures the elimination of damaged or abnormal germ cells, preserving only the healthy ones for the production of viable sperm (Shaha et al., 2010). Our results suggest that exposure to cryoprotective solutions (DMSO and sucrose), as well as the thawing temperature (37 °C) and the three-dimensional in vitro culture (IVC) system, modulate the expression of pro‑apoptotic genes in testicular fragments, indicating potential alterations in the testicular microenvironment.

Scanning and transmission electron microscopy analyses demonstrated increased structural damage in testicular tissue following in vitro culture, consistent with the general morphological findings, irrespective of preservation time or cryopreservation. Previous studies in humans (Keros et al., 2005; Portela et al., 2019), mice (Lu et al., 2022), and goats (Gomes et al., 2023) reported greater damage to the testicles after *in vitro* culture. The ultrastructure allowed for more detailed visualization of cellular structure, identifying possible damage to the membrane, organelles, and other intracellular structures, such as vacuolization and cell loss resulting from the cryopreservation and *in vitro* culture processes, which were not observed in the testicular fragments that were only conserved and cryopreserved. This suggests that our protocol was suitable for conserving and cryopreserving prepubertal goat testicles.

## 5. Conclusion

Based on the results obtained in this study, we concluded that the appropriate definition of testicular preservation time at 4 °C before slow-freezing procedures and/or in vitro culture is a critical factor for the success of these biotechnological strategies. Control of this interval directly affects testicular tissue viability and quality, thereby influencing the success of reproductive function preservation. In addition, the information presented herein may guide technicians and producers in decision-making when testicular recovery and handling are required, particularly regarding cryostorage in germplasm banks of genetically valuable animals. These findings are especially relevant in situations involving unexpected and irreversible losses, contributing to the adoption of assisted reproductive biotechnologies and even to biodiversity conservation.

## Data Availability

Research data is available in the body of the article.
